# Supplementary material for: Three-photon excited fluorescence microscopy enables imaging of blood flow, neural structure and inflammatory response deep into mouse spinal cord in vivo
Source: bioRxiv. 2024 Apr 6:2024.04.04.588110. Preprint. [Version 1] doi: 10.1101/2024.04.04.588110 (PMC11014502; doi:10.1101/2024.04.04.588110)
Supplement: 1 [file NIHPP2024.04.04.588110V1-supplement-1.pdf]

bioRxiv preprint doi: https://doi.org/10.1101/2024.04.04.588110; this version posted April 6, 2024. The copyright holder for this preprint (which was not certified by peer review) is the author/funder, who has granted bioRxiv a license to display the preprint in perpetuity. It is made available under aCC-BY 4.0 International license.

598 **Supplementary Materials**

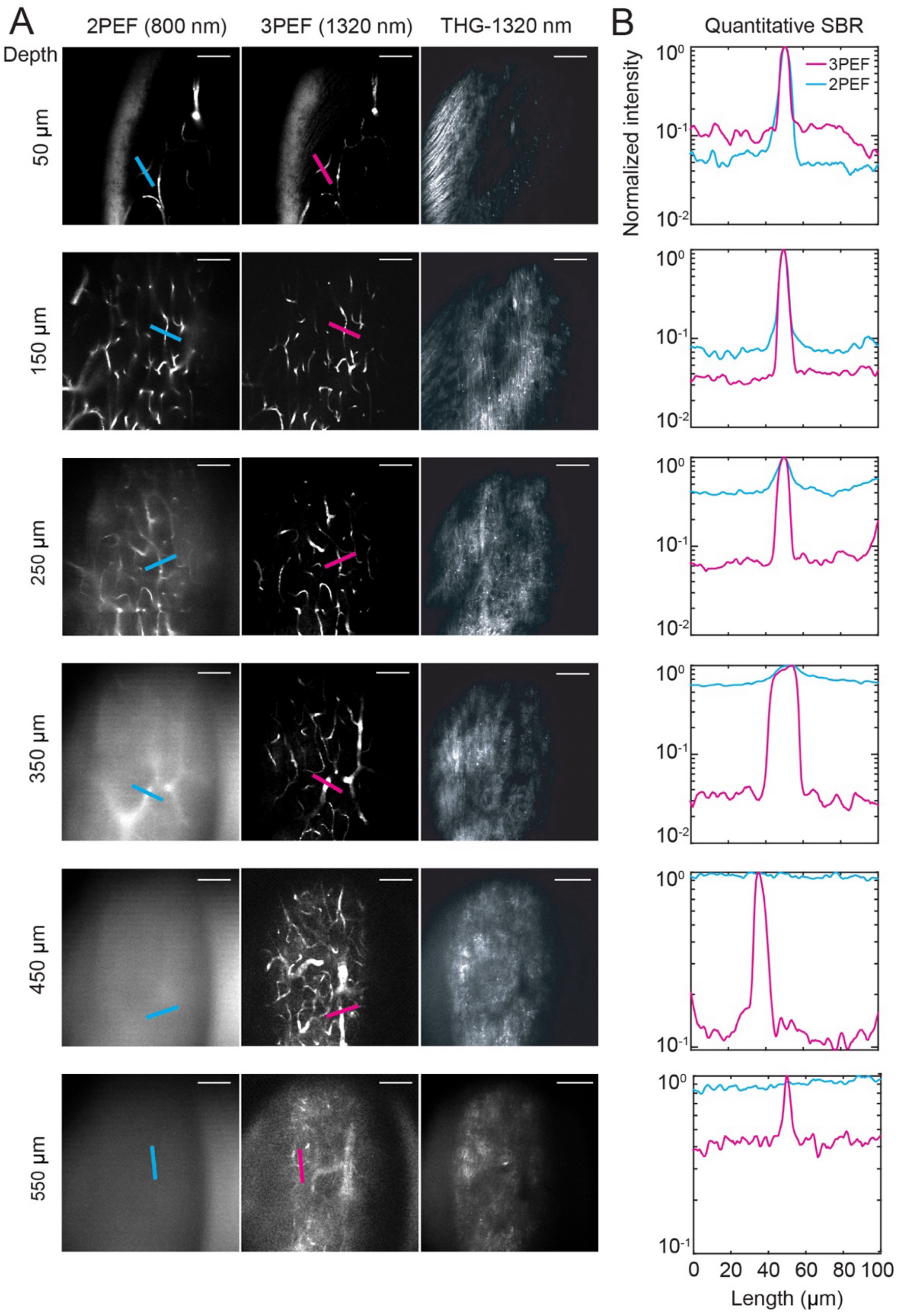

599

600 **Figure 1—figure supplement 1. Representative images of fluorescein-labeled vasculature**
601 **stack under 2PEF at 800-nm versus 3PEF at 1320-nm excitation source.** (A) 2PEF (left
602 column), 3PEF (middle column), and THG images (right column) at selected depths into the
603 mouse spinal cord. Scale bar: 100 µm. (B) Line profiles across selected capillaries, comparing
604 3PEF (magenta) and 2PEF (blue) image contrast at different depths. Lines in (A) indicate

bioRxiv preprint doi: https://doi.org/10.1101/2024.04.04.588110; this version posted April 6, 2024. The copyright holder for this preprint (which was not certified by peer review) is the author/funder, who has granted bioRxiv a license to display the preprint in perpetuity. It is made available under aCC-BY 4.0 International license.

location of lineouts. These data include the three depths shown in Figs. 1C and D and add three additional depths. Scale bar: 100 μm.

bioRxiv preprint doi: https://doi.org/10.1101/2024.04.04.588110; this version posted April 6, 2024. The copyright holder for this preprint (which was not certified by peer review) is the author/funder, who has granted bioRxiv a license to display the preprint in perpetuity. It is made available under aCC-BY 4.0 International license.

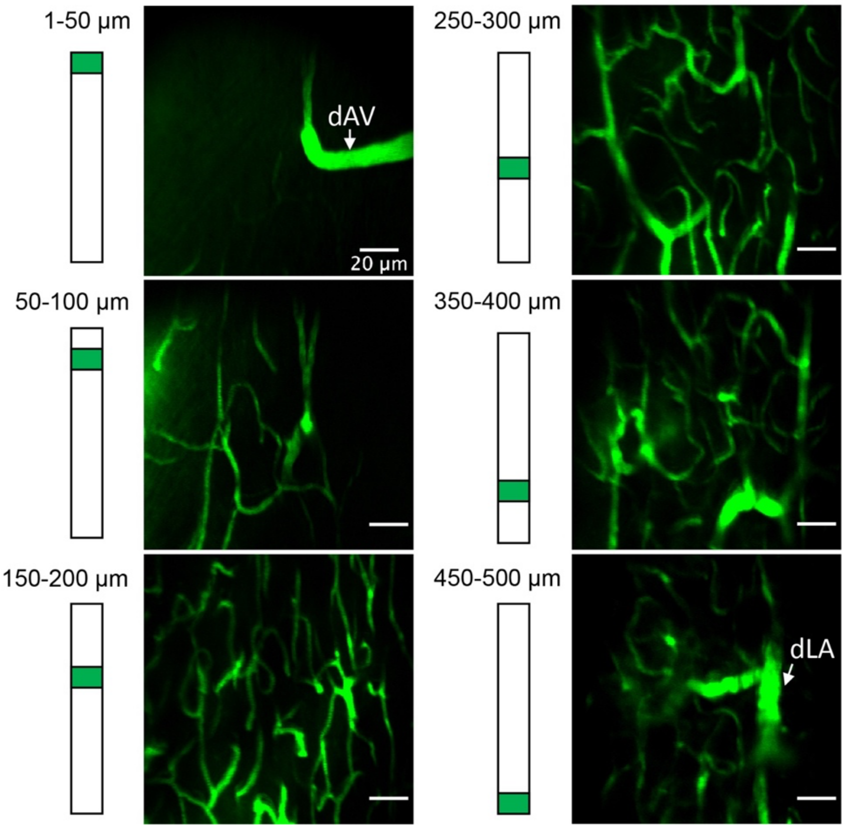

Figure 2—figure supplement 1. Representative images demonstrating spinal cord vasculature, including dAV at the surface and dLA at depth. Selected images from a continuous vasculature stack indicate the dAV at the top 50 μm followed by a dense capillary network spanning across the spinal cord tissue column, which is fed by dLA that runs along the rostral-caudal axis of the mouse spinal cord.

bioRxiv preprint doi: https://doi.org/10.1101/2024.04.04.588110; this version posted April 6, 2024. The copyright holder for this preprint (which was not certified by peer review) is the author/funder, who has granted bioRxiv a license to display the preprint in perpetuity. It is made available under aCC-BY 4.0 International license.

Movie S1. Images stacks showing spinal cord vasculature taken with 2PEF (left) vs 3PEF (right). Stacks correspond to the image data in Figure 1.

Movie S2. Three-dimensional rendering of 3PEF image stack from Thy1-YFP x Cx3Cr1-GFP mouse showing axons (YFP, shown in green) and microglia (GFP, shown in magenta). Image stack corresponds to data shown in Figure 3B, taken at baseline, before the dAV occlusion.

Movie S3. 3PEF image stacks of blood vessels (Qtracker^{TM} 655, shown in red), axons (YFP, shown in yellow), and myelin (THG, shown in greyscale) taken at baseline, and at 30 min, 60 min, and 120 min after the occlusion of a dorsal ascending venule. Image stacks correspond to the occlusion site shown in Figure 3C.
